# Do University Clinical Aptitude Test (UCAT) scores predict doctors’ performance in UK post-qualification practical clinical examinations? A protocol for an observational retrospective national cohort study

**DOI:** 10.1136/bmjopen-2026-117049

**Published:** 2026-09-01

**Authors:** Taha Khan, Lewis William Paton, Paul Alexander Tiffin

**Affiliations:** 1 University of York, York, UK

**Keywords:** Education, Medical, Psychometrics, Clinical Competence, Health Workforce

## Abstract

**Abstract:**

**Introduction:**

Internationally, medical schools increasingly use standardised selection assessments to select applicants. The University Clinical Aptitude Test (UCAT) is the most popular standardised medical selection assessment across Europe and Australasia. The UCAT consists of cognitive assessments and a situational judgement test. The UCAT scores have been shown to offer incremental predictive validity for medical school academic attainment: they add predictive value beyond that already provided by prior educational attainment such as secondary school grades. However, the use of standardised medical selection assessments imposes burdens on applicants and institutions. Therefore, to justify their widespread adoption, scores from these assessments should predict doctors’ future clinical competency. This can be evaluated by their performance in post-qualification practical clinical examinations. Hence, this study aims to evaluate whether UCAT scores offer incremental predictive validity for doctors’ performance in UK post-qualification practical clinical examinations.

**Methods and analysis:**

This observational retrospective national cohort study will use data from the United Kingdom Medical Education Database (UKMED). The UKMED is a national database containing pseudo-anonymised routine data on UK doctors. The population included will be those who attempted the UCAT as part of their medical school application and subsequently attempted UK post-qualification practical clinical examinations. The predictors are the UCAT total and subtest scores. The primary outcomes are performance in the UK post-qualification practical clinical examinations, such as the Membership of the Royal College of Physicians Practical Assessment of Clinical Examination Skills (MRCP PACES). The sample size estimate is >270.

Descriptive statistics will summarise the study data. Univariable logistic regression will estimate the probability of passing post-qualification practical clinical examinations as a binary categorical outcome variable (pass vs fail), based on UCAT scores. Multivariable logistic regression will estimate whether UCAT scores offer incremental predictive validity for passing post-qualification practical clinical examinations by adjusting for the predictive validity offered by prior educational attainment. Similarly, univariable and multivariable linear regression will estimate the predictive validity of UCAT scores for performance in post-qualification practical clinical examination scores as a continuous interval variable. Path analyses will be developed and tested to explore how the educational predictors relate to each other and the outcomes of interest. Missing data will be addressed using multiple imputation by chained equations.

**Ethics and dissemination:**

As this is a secondary analysis of pseudo-anonymised data, the Chair of Hull York Medical School Ethics Committee confirmed in writing that ethical approval will not be required. Results will be published in a peer-reviewed journal, presented at academic conferences, and shared with relevant policy bodies.

**Registration:**

The protocol was registered prospectively on the Open Science Framework (10.17605/OSF.IO/VZ2QC).

**STRENGTHS AND LIMITATIONS OF THIS STUDY**

- A large national dataset (UKMED), with comprehensive coverage of UK medical graduates, will enhance external validity and generalisability.
- Important methodological limitations, identified in previous research, will be addressed by using a representative multi-institutional sample, accounting for the full range of prior educational attainment, and appropriately handling missing data through imputation.
- Complementary statistical approaches will be used, including univariable and multivariable logistic and linear regression to assess incremental predictive validity, and path analysis to examine the intervariable relationships and mechanisms by which University Clinical Aptitude Test (UCAT) scores predict post-qualification clinical examination performance.
- The primary outcome of post-qualification practical clinical examination performance will provide a valid proxy for clinical competency that is standardised and directly relevant to patient care.
- The observational design limits causal inference, and the study is restricted to UK data, which may limit generalisability to other healthcare systems and selection assessments.

## Introduction

 The selection of future doctors is critically important due to the personal and institutional investment^1^, and the trust society places in the role.^2^ Internationally, medical selection remains a challenging endeavour due to the high volume of similarly academically high-achieving candidates. In addition, there are important ethical considerations as pre-university educational attainment is closely associated with socioeconomic status.^3^ To address the practical and ethical challenges of medical selection, medical schools have increasingly adopted standardised selection assessments. They produce scalar metrics (scores) of performance, which are then used by selectors in a variety of ways to support and defend their admissions decisions.^4 5^

The University Clinical Aptitude Test (UCAT) is the most popular standardised medical selection assessment across Europe and Australasia. After evolving over the years, it now includes different cognitive tests and a situational judgement test (SJT).

Despite their international adoption, standardised medical selection assessments have faced substantial criticism. First, concerns have been raised regarding: their fairness; the added financial burden, time and stress imposed on applicants; and their limited relevance to real-world medical practice.^6–9^ Second, although they were intended to widen access to medicine,^10^ the evidence suggests that the use of these tests did not have a causal impact on increasing medical school admissions from underrepresented groups.^11 12^ Third, serious errors have occurred in their administration.^13^

There is already some evidence that the scores from such selection assessments can, at least modestly, predict academic performance during medical school, incremental to the prediction offered by prior educational attainment, such as secondary (high) school grades.^14–18^ However, individuals are admitted to medical school not just to study medicine, but to practise it. Thus, to justify their international adoption, given the drawbacks above, the scores from standardised medical selection assessments should demonstrate predictive validity for markers of doctors’ post-qualification clinical competency.^19–21^

Following their review, Epstein and Hundert defined clinical competency as *the habitual and judicious use of communication, knowledge, technical skills, clinical reasoning, emotions, values, and reflection in daily practice for the benefit of the individual and the community being served*.^22^ Therefore, in an ideal scenario, objective measures of clinical outcomes would be the preferred validity criteria for clinical competency. However, obtaining such data is extremely challenging. Moreover, it is difficult to account for the effects of potential confounding variables, such as access to resources and workload. Hence, performance in post-qualification practical clinical examinations is likely the best readily available proxy for an individual doctor’s clinical competency. These examinations typically involve high-fidelity clinical simulations that are structured and standardised, at least to some extent, in scoring and content. Furthermore, there is strong evidence that a doctor’s performance in post-qualification clinical examinations predicts actual clinical outcomes, such as mortality rate^23 24^ and sanctions on a doctor’s licence to practise.^25 26^

A recent, but not yet published, systematic review found evidence that scores from standardised medical selection assessments, including the UCAT, can provide modest incremental predictive validity for doctors’ performance in post-qualification practical clinical examinations. Nevertheless, it remains unclear how the predictive validity of UCAT scores varies: (1) depending on the medical specialty (such as physicians vs surgeons) or (2) if the UCAT’s SJT scores are also taken into consideration. Furthermore, the limited existing literature has some methodological limitations due to non-representative and/or single-institution samples as well as not accounting for the full range of prior educational attainment, and the inevitable range restriction, right censoring and missing data.

Therefore, this observational retrospective national cohort study aims to address the important knowledge gaps and methodological limitations identified in the systematic review by investigating whether UCAT scores offer predictive validity for doctors’ performance in UK post-qualification practical clinical examinations.

## Methods and analysis

### Patient and public involvement

Patients and the public will not be directly involved in the conduct, reporting or dissemination of this study. However, they were involved in shaping the focus of this project. Through engagement with key stakeholders, including medical educators, doctors, policymakers and patient representatives, we confirmed that improving medical selection is a priority given the resource-intensive and demanding nature of medical education.^27 28^ Specifically, it would be helpful to know if standardised medical school selection assessments predict markers of doctors’ clinical competency.

### Study design and protocol

This is an observational retrospective national cohort study. The protocol was registered prospectively on the Open Science Framework (10.17605/OSF.IO/VZ2QC).

### Data source

The United Kingdom Medical Education Database (UKMED) is a national database which contains pseudo-anonymised routinely collected data on the demographics, career choices and educational attainment of UK doctors before, during and after medical school. UKMED data will be accessed via the University of Dundee’s Health Informatics Centre (HIC) Trusted Research Environment (TRE), which is a secure computing environment. This means the raw data can only be accessed by approved users within their platform.

### Study population

Since the data source is the UKMED, the population for this study was doctors who graduated from a UK medical school and attempted the updated version of the UCAT, which included SJT, from 2013 to 2022. The date of extraction was 31 January 2025. Further details about the eligibility criteria are available in table 1.

**Table 1:** Inclusion and exclusion criteria

Inclusion criteria	Exclusion criteria
Doctors who: Attempted the updated UCAT, which includes the SJT, as part of their application to medical school, independent of whether their medical school used UCAT scores to inform admissions decisions. Successfully graduated from medical school. Subsequently attempted a UK post-qualification practical clinical examination, independent of whether they passed or failed.	Candidates who attempted the UCAT but did not successfully graduate from medical school.

SJT, situational judgement test; UCAT, University Clinical Aptitude Test.

### Power analysis

Since this was a secondary analysis of existing data, the sample size was fixed by the availability of records in the UKMED. Hence, the smallest detectable effect size was calculated to inform the interpretation of findings which are not statistically significant: these may represent a type II error (failing to detect a true association because the sample was not big enough), rather than a genuine absence of association.

The smallest detectable effect size for our primary analysis, the logistic regression of the odds of first attempt pass (see the Regression analysis section), was calculated using the method by Hsieh, Bloch and Larsen.^29^ The alpha (level of statistical significance) was chosen to be 0.05, in line with established practice in medical research, thereby controlling the type I error rate at a maximum of 5%. The beta (type II error rate) was chosen to be 0.2, corresponding to a statistical power of 80%. This means that, if a true association of the specified magnitude exists, the analyses would be expected to detect it in 80% of repeated samples drawn under the same conditions. A one-sided test, or tail, was used as the theoretical premise,^30^ and existing data^21 31^ demonstrate that a negative relationship between the predictor and outcome would be very unlikely. Based on stakeholder engagement, and review of the pass rates of some recent post-qualification practical clinical examinations, we concluded that a pass rate of two-thirds is reasonable to use.

Therefore, the smallest detectable OR was calculated to be approximately 1.5 per SD increase in UCAT total score: 1.524 for predictive validity (using UCAT scores as the predictor) and 1.540 for incremental predictive validity (using UCAT scores as the predictor but adjusting for prior school attainment). An OR of 1.5 also represents a reasonable threshold for the smallest effect of practical importance. This is because a one SD increase in UCAT total score is itself substantial: assuming UCAT scores are normally distributed, it moves a candidate from the 50th to the 84th percentile. At a first attempt pass rate of two-thirds, the odds of passing are 2 to 1. An OR of 1.5 raises these odds to 3 to 1. While the change in odds seems large, practically, this is only 8 more passes per 100 candidates (75% vs 67%). Hence, we may be able to conclude that if a statistically significant OR is not detected, the actual OR is likely too small to be of practical importance.

Unlike the regression analyses, an a priori power analysis for the path models (see the Path analysis section) would be more challenging as it depends on: the number of parameters estimated; the model structure; and the degree to which the hypothesised model structure reflects the observed relationships in the data. Hence, it will not be attempted, which is a limitation of this study. Nevertheless, following the final path model specification, a post-hoc power analysis using Monte Carlo simulation will be considered, if practical.^32^ This approach works by repeatedly generating simulated datasets based on plausible variations in the estimated model parameters, then fitting the path model to each simulated dataset. By examining how often the model recovers the pre-specified parameter values across the simulations, it provides a post-hoc estimate of whether the available sample was sufficient to detect effects of the magnitude observed. Additionally, examining how much the recovered parameter estimates vary across simulated datasets indicates their stability.

### Predictors

The predictors chosen were those required to answer the research question and those which were most important for policy and for stakeholders, including medical school selectors and the funder. These included the UCAT total scores and subtest scores (verbal reasoning, quantitative reasoning, decision analysis, abstract reasoning, *a*nd the SJT) as well as prior educational attainment, such as A-level grades. The UCAT cognitive scores are available as z-scores, standardised according to all test-takers at that sitting. Other predictors included medical school performance, foundation programme SJT scores, and other UK post-qualification medical examinations, such as the multi-speciality recruitment assessment. These other predictors will be included to inform path analysis and facilitate the identification of indirect prediction mechanisms (see the Path analysis section).

The first attempt examination scores will be used, with subsequent attempts excluded from analysis, to mitigate against practice effects, and use the most valid assessment of underlying ability. Furthermore, the standardised (z) scores will be used, according to all test-takers, where possible, to: facilitate comparison between different test years, accounting for possible changes in test difficulty; calculate incremental predictive validity; and address range restriction (candidates with low UCAT scores are unlikely to be admitted to medical school).

### Outcomes

The primary outcomes of interest are performance in UK post-qualification practical clinical examinations, such as MRCP PACES. Again, the first attempt examination scores will be used, with subsequent attempts excluded from analysis, to mitigate against practice effects and use the most valid assessment of underlying ability. Furthermore, the relative-to-pass (RtP) scores will be used to facilitate comparison between different test years, accounting for possible changes in test difficulty, to calculate incremental predictive validity.

### Data management

Data will be managed, analysed and produced using Stata V.17. Data cleaning is an example of data management and will address data that are incomplete, incorrect or irrelevant to the task at hand. Data cleaning will include: removing unwanted data, such as duplicates; renaming variables for consistency and clarity; standardising variable formats; and identifying and addressing outliers or implausible values.

Data management will also involve merging and reshaping datasets, preparing them for the planned analyses. Merging will involve combining the UCAT scores with post-qualification examination results. Reshaping will involve adapting datasets between wide format (where each doctor has one row with multiple examination scores in separate columns) and long format (where each doctor has multiple rows with one row per examination).

### Descriptive statistics

Descriptive statistics will summarise the study data, identify potential data quality issues requiring further cleaning, characterise patterns of missingness, and provide a foundation for subsequent inferential statistics, such as regression analysis. For categorical variables, such as first-attempt pass or fail in post-qualification practical clinical examinations, frequencies and percentages will be tabulated. For continuous variables, such as the UCAT scores and post-qualification practical clinical examination scores (RtP), measures of central tendency (mean, median) and variability (SD, minimum, maximum) will be calculated.

Visual inspection of distributions will be facilitated by histograms and quantile-quantile (Q-Q) plots. Histograms will display the frequency of values across the range, revealing whether data are symmetrically or asymmetrically distributed (ie, skewed), and identifying any extreme or implausible values. Q-Q plots will compare the observed data against a theoretical normal distribution, which helps clarify whether the observed data are normally distributed. This informs the choice of correlation method: the Pearson product-moment correlation coefficient for normally distributed data versus the Spearman rank-order correlation coefficient for non-normal distributions. Then, a correlation matrix will check for multicollinearity between the variables (see the Methodological limitations section).

### Regression analysis

Regression analysis will be used to estimate how changes in an independent (predictor) variable are associated with changes in a dependent (outcome) variable. Logistic regression is a type of regression analysis that uses maximum likelihood estimation to estimate how a change in a predictor relates to a change in a binary categorical outcome (choice between two categories, such as ‘pass’ or ‘fail’). It works by transforming predictor variables, through a logistic function, to calculate the probability of each observed outcome (between 0 and 1), then iteratively identifies coefficients for the predictors, resulting in the maximum probability that the observed outcomes occurred, across all observations in the dataset.^33^

Here, univariable logistic regression will primarily be used to estimate the probability that a doctor passes their post-qualification practical clinical examination, on their first attempt, based on their total and subtest UCAT z-scores. Logistic regression will serve as the primary analysis as pass/fail outcomes are the most important for workforce policy since they are instrumental in determining doctors’ career progression. Logistic regression will also be applied to the other aforementioned predictors, such as medical school performance, to inform subsequent path analysis and facilitate the identification of indirect prediction mechanisms (see the Path analysis section).

To evaluate the practical importance of the logistic regression findings, the C-statistic and Number Needed to Reject (NNR) will be reported. The C-statistic (area under the curve) is a measure of discrimination that assesses how well the logistic regression model distinguishes between candidates who pass their post-qualification practical clinical examination on their first attempt, from those who fail. It is interpreted as the probability that a randomly selected candidate who passed scored higher on the UCAT than a randomly selected candidate who failed. Thus, a value of 0.5 indicates a prediction no better than random chance (50:50), while a value of 1.0 indicates perfect discrimination. The NNR is the total number of candidates who would need to be rejected, at a given UCAT score threshold, including those who would have passed their respective post-qualification practical clinical examination, to reject one candidate who would have failed.^16 25^ Hence, a lower NNR indicates a more effective screening threshold. This is directly relevant to admissions policy, as many universities use UCAT scores as a threshold for inviting candidates to interview. Furthermore, it is more easily interpretable for our target audience, which is university selectors and policymakers, who may not have a background in statistics.

To assess incremental predictive validity, multivariable logistic regression will be used. This will involve comparing two nested models: a base model containing only non-UCAT educational attainment (such as A-level grades), and a full model that additionally contains the total UCAT z-score. The incremental predictive validity of total UCAT z-scores will be assessed by using a likelihood ratio test to determine whether the improvement in model fit, between the base model and the full model, is statistically significant. The above steps will be repeated for all the individual UCAT cognitive subtest z-scores and UCAT SJT scores.

Linear regression is another type of regression analysis that examines how predictor variables (such as UCAT scores and prior educational attainment) relate to a continuous outcome variable (post-qualification clinical examination performance, relative to pass mark). Both linear regression and logistic regression assume a linear relationship: in linear regression, changes in predictor variables are associated with proportional changes in the outcome; whereas, in logistic regression, changes in predictor variables are associated with proportional changes in the natural logarithm of the odds (log odds). Linear regression works by finding the coefficients of the predictors that result in the minimum sum of the squared differences between the observed and predicted outcomes.^34^ Linear regression will serve as the secondary analysis as continuous RtP scores provide finer discrimination of performance, complementing the binary pass/fail prediction offered by logistic regression. Linear regression will similarly be applied to the other aforementioned predictors, such as medical school performance, to inform subsequent path analysis and facilitate the identification of indirect prediction mechanisms (see the Path analysis section). The approach for assessing incremental predictive validity using linear regression will mirror the approach using logistic regression. Incremental R^2^ will quantify the additional proportion of variance in post-qualification practical clinical examination performance, explained by using the full model instead of the base model.

### Path analysis

Path analysis is a statistical technique that models the linear relationships between different variables. It helps distinguish between direct effects (where one variable predicts another without intermediary steps) and indirect effects (where one variable predicts another through a mediating variable).^35^ This distinction is important because it clarifies the mechanisms by which UCAT scores may predict doctors’ performance in post-qualification clinical examinations. For example, if the multivariable linear regression shows that UCAT scores do not offer incremental predictive validity for post-qualification practical clinical examination performance, university selectors may be inclined to stop using them. However, the path analysis may reveal that, while there is not a direct effect (ie, incremental predictive validity) between UCAT score and post-qualification practical clinical examination performance, an indirect mediated predictive effect may exist. For example, UCAT scores may instead directly predict medical school performance, which directly predicts post-qualification practical clinical examination performance. Therefore, while regression analyses will facilitate an understanding of the predictive validity, path analysis will clarify the mechanisms. Path analysis will serve as the tertiary analysis as it is contingent on findings from the primary logistic and secondary linear regression analyses, which will inform the specification of direct and indirect pathways in the path models. Path analysis also accommodates the temporal ordering and continuity of educational predictors in the model.

**Figure 1:**
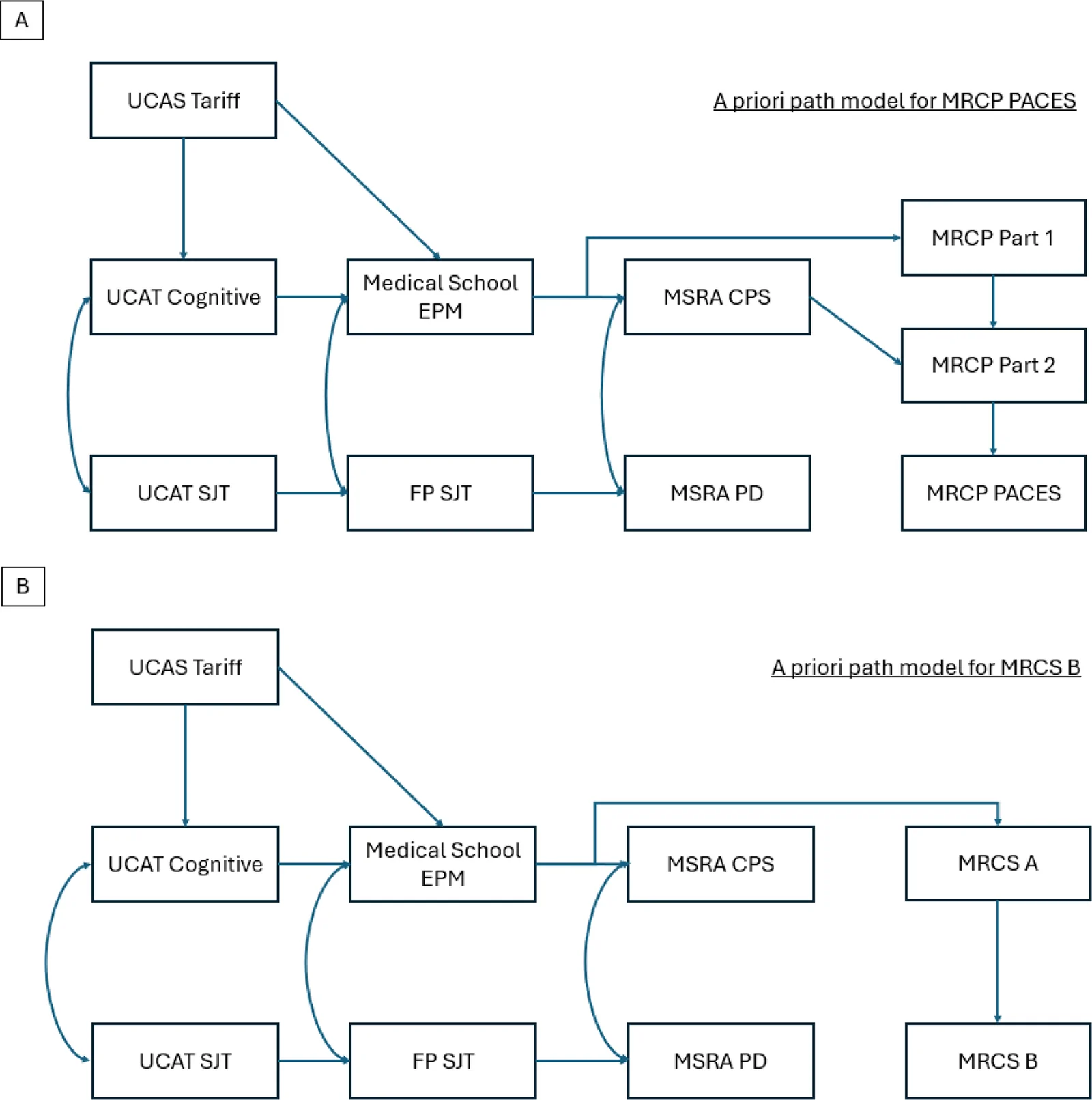
A priori path models for (**A**) MRCP PACES and (**B**) MRCS B. CPS, Clinical Problem Solving; EPM, Educational Performance Measure; FP, Foundation Programme; MRCP PACES, Membership of the Royal College of Physicians Practical Assessment of Clinical Examination Skills; MRCS B, Membership of the Royal College of Surgeons B; MSRA, Multi-Specialty Recruitment Assessment; SJT, Situational Judgement Test; UCAS, Universities and Colleges Admissions Service; UCAT, University Clinical Aptitude Test.

However, a key limitation of path models is that the hypothesised structure needs to be correctly specified in advance of the analysis.^36^ Therefore, the proposed path models (figure 1) were informed by expert opinion and the existing literature. For example, initial path models were discussed with several resident doctors who were surgeons and physicians, with recent lived experience of sitting these assessments. Path coefficients will quantify the strength of each pathway. Total effects will be decomposed into their direct and indirect components. Model fit will be evaluated using established indices, such as the Comparative Fit Index, Tucker-Lewis Index, and root mean square error of approximation.^37^ If a priori path models do not adequately fit the data, stepwise modifications will be performed, taking into account the modification indices, and evidence of localised ‘model strain’, including the magnitude of the estimated path coefficients. These can be iteratively refined, informed by stakeholder feedback, preserving theoretical plausibility. Our strategy for reconciling potentially conflicting results between our primary, secondary and tertiary analyses will be narrative, based on the methodological differences in these approaches.

### Missing data

Missing data refers to values that should have been recorded but are absent from the dataset.^38^ For example, if prior educational attainment is not available for some participants. Missing data can occur through different mechanisms: missing completely at random, where missingness is unrelated to any variables; missing at random (MAR), where missingness depends on observed variables but not on the missing values themselves; or missing not at random (MNAR), where missingness depends on the unobserved missing values.^38^

The consequences of unaddressed missing data include: reduced statistical power (lower probability of detecting the true effect); biased results (the observed relationships are systematically different from the true relationships); and loss of precision in estimates (wider CIs leading to greater uncertainty).^39^ To circumvent these limitations, missing data will be addressed using imputation, rather than complete case analysis (analysing only participants with complete data).

Imputation will be informed by the missingness revealed from descriptive statistics. Nevertheless, the plan is to use multiple imputation by chained equations (MICE). MICE is a method of imputation with several advantages since it: incorporates all available information from the dataset to enhance prediction of missing values; introduces random variation in the imputed estimates, reflecting the uncertainty from the observed relationships; generates multiple data sets, reflecting the between-imputation uncertainty; and then analyses and pools the results, accounting for the total uncertainty in the final estimates.^40^

MICE will be used to impute both predictor variables (such as prior educational attainment) and outcome variables (performance in post-qualification practical clinical examinations). Imputing outcome data addresses right censoring, where some doctors had not yet attempted or completed a postqualification practical clinical examination by the time of analysis, and mitigates range restriction that would occur by excluding these doctors from analysis. For linear and logistic regression analyses, results will be pooled across the m imputed datasets.^38^

MICE is appropriate to use for predictors as it is reasonable to assume that data are MAR: missingness in this dataset is likely to depend on observed variables (such as cohort year or examination type), rather than the unobserved values themselves. Similarly, since this dataset contains multiple measures of ability, outcome missingness could also be due to observed variables rather than unobserved values. However, it could be argued that outcome missingness also has an MNAR component: brighter and less risk-averse candidates attempt these examinations earlier in their careers, while others delay until they feel adequately prepared due to the high cost and emotional toll of failing. Nevertheless, even if the MAR assumption does not fully hold, and some degree of MNAR exists, imputation with sensitivity analyses represents the best available approach to address right censoring and range restriction caused by missing outcome data, providing better insights than complete-case analysis alone. Imputed analysis will inform construct-level predictive validity: whether the constructs (or cognitive abilities) measured by the UCAT subtests predict the doctors’ clinical competency.^41^ In contrast, complete-case analysis of non-imputed data will answer the more policy-relevant question: whether the UCAT scores themselves predict the performance of doctors who attempt their post-qualification practical clinical examinations. Comparison between these two estimates will reveal the extent to which range restriction attenuates observed correlations.

In addition to the main imputed analysis and the complete-case analysis, a further sensitivity analysis will be conducted in which the predictors are imputed but not the outcomes. Hence, only doctors who actually attempted a post-qualification practical clinical examination will be retained. This will isolate the effect of imputing the outcomes from the effect of imputing the predictors. Therefore, it would answer the same policy-relevant question as the complete-case analysis, namely whether UCAT scores predict performance among doctors who attempt these examinations, but will do so using the full set of such doctors rather than only those with complete predictor data. Comparing this sensitivity analysis with the complete-case analysis will show whether the complete-case estimates were affected by missing predictor data. In contrast, comparing the sensitivity analysis with the main imputed analysis will show how much of the difference between the two is attributable to imputing the outcomes in addition to the predictors.

The number of imputations will be decided by the point at which the imputed results stabilise. In practice, this is likely to be between m=20 and m=30 imputations. Thus, it is likely to meet the threshold suggested by methodological guidance of at least 20 or equal to the percentage of participants with missing values, whichever is greater.^42^

### Limitations

This study will have limitations, due to the data and methods, that should be considered when interpreting the findings.

#### Data limitations

The data limit generalisability for many reasons. First, smaller and more niche specialities will have insufficient numbers to adequately power analyses. Therefore, the findings may not generalise to all specialties. Second, the data are from historical cohorts. Thus, the findings may not generalise to current or future cohorts. This is particularly relevant as selection criteria and assessments change over the years. Finally, the data are based on doctors in the UK who attempted the UCAT and UK post-qualification practical clinical examinations. Hence, the findings may not generalise to other standardised medical selection assessments, international medical graduates or other healthcare systems.

#### Methodological limitations

Firstly, the observational retrospective cohort design limits causal inference about the relationship between UCAT scores and doctors’ post-qualification clinical examination performance. While *associations* can be identified (ie, predictive validity), the study cannot definitively establish whether better UCAT performance *causes* better clinical examination performance; however, the latter is not the goal of the study.

Second, performance during a high-stakes clinical examination may not always reflect behaviour in routine, daily practice.

Third, many unmeasured plausibly confounding variables exist, such as non-cognitive attributes, socioeconomic factors or clinical experience. These may influence both UCAT scores and clinical examination performance. Unfortunately, these cannot readily be controlled for. Hence, results may be biased.

Furthermore, both linear regression and path analysis have limitations as they assume: linearity (changes in predictor variables are associated with proportional changes in the outcome); normality of errors (the difference between the predicted value and actual value (ie, the ‘error’) is normally distributed); homoscedasticity (the variance of the errors should be consistent across all levels of the predicted values) and absence of multicollinearity (predictor variables should not be too highly correlated with each other). While all these assumptions are present in both linear regression and path analysis, the latter has an additional unique assumption that the hypothesised structure is correctly specified without missing variables.^36^ In addition, the path analyses may not be powered.

## Ethical considerations

The Medical Act 1983 gives the General Medical Council (the UK medical regulator) a legal responsibility to promote high standards of medical education and coordinate all stages of medical education.^43^ They use this to justify the UKMED.

Since the data are anonymised, separate ethical approval is not required under current UK governance frameworks. This was confirmed in writing by the Chair of Hull York Medical School Ethics Committee. Furthermore, individual consent was not required under the UK General Data Protection Regulation statutory functions provision. In addition, as described in *Data Source*, data will be accessed via the HIC TRE, which, according to their website, ‘is specifically designed for handling sensitive data in a way that protects privacy and ensures security’ and prevents the extraction of individual data. Finally, results will be presented in an aggregated, blunted form, preventing individual recognition, in keeping with the Higher Education Statistics Agency guidelines.^44^

## Timeline

Data cleaning should take place from June to August 2026. Statistical analysis should take place from August to October. Hence, the manuscript should be written from October to December 2026.

## Dissemination

The findings from this study will be disseminated through multiple channels to maximise impact.

### Open science practices

The study protocol has been pre-registered on the Open Science Framework (OSF), ensuring transparency and reducing the risk of selective reporting. Raw data may be made available on application to the UKMED citing project *UKMEDP181*. Code will be made publicly available via GitHub following publication, inviting reproducibility and methodological scrutiny. The study will follow Strengthening the Reporting of Observational Studies in Epidemiology (STROBE) guidelines^45^ for transparent reporting of observational research.

### Academic outputs

The manuscript will be submitted for publication in a high-impact, peer-reviewed medical education journal, with open access publication prioritised, where funding permits, to ensure broad accessibility. Abstracts will also be sent to national or international medical education conferences.

### Policy engagement

Findings will be shared with: the UCAT Board, which oversees the development and administration of the UCAT across participating medical schools, to inform future test development; the Medical Schools Council Selection Alliance, which provides guidance on medical school admissions practices across the UK; and relevant Royal Colleges that administer post-qualification clinical examinations to better inform their understanding of predictors of performance in their assessments.

## References

[1] Yates J (2012). When did they leave, and why? A retrospective case study of attrition on the Nottingham undergraduate medical course. BMC Med Educ.

[2] Clemence M (2022). Doctors and scientists are seen as the world’s most trustworthy professions. https://www.ipsos.com/en-uk/doctors-and-scientists-are-seen-worlds-most-trustworthy-professions.

[3] Mwandigha LM, Tiffin PA, Paton LW (2018). What is the effect of secondary (high) schooling on subsequent medical school performance? A national, UK-based, cohort study. BMJ Open.

[4] Kreiter CD, Axelson RD (2013). A perspective on medical school admission research and practice over the last 25 years. Teach Learn Med.

[5] Greatrix R, Dowell J (2020). UKCAT and medical student selection in the UK - what has changed since 2006?. BMC Med Educ.

[6] Dhar D, Perry WRG, Poole P (2012). Students’ perceptions of the Undergraduate Medicine and Health Sciences Admissions Test (UMAT). N Z Med J.

[7] Fortin Y, Kulasegaram KM, Kancir JN (2018). The Direct Economic and Opportunity Costs of the Medical College Admissions Test (MCAT) for Canadian Medical Students. MedEdPublish (2016).

[8] Kelly ME, Patterson F, O’Flynn S (2018). A systematic review of stakeholder views of selection methods for medical schools admission. BMC Med Educ.

[9] Kumar K, Roberts C, Bartle E (2018). Testing for medical school selection: What are prospective doctors’ experiences and perceptions of the GAMSAT and what are the consequences of testing?. Adv Health Sci Educ Theory Pract.

[10] Greatrix R (2022). What impact has the United Kingdom clinical aptitude test (UKCAT) had on selection to undergraduate medicine and dentistry in the UK?. https://eprints.nottingham.ac.uk/71714/1/Thesis%20%28final%29.pdf.

[11] Mathers J, Sitch A, Parry J (2016). Longitudinal assessment of the impact of the use of the UK clinical aptitude test for medical student selection. Med Educ.

[12] Fielding S, Tiffin PA, Greatrix R (2018). Do changing medical admissions practices in the UK impact on who is admitted? An interrupted time series analysis. BMJ Open.

[13] Cassidy J (2008). UKCAT among the pigeons. BMJ.

[14] Dunleavy DM, Kroopnick MH, Dowd KW (2013). The predictive validity of the MCAT exam in relation to academic performance through medical school: a national cohort study of 2001-2004 matriculants. Acad Med.

[15] Puddey IB, Mercer A (2014). Predicting academic outcomes in an Australian graduate entry medical programme. BMC Med Educ.

[16] Tiffin PA, Mwandigha LM, Paton LW (2016). Predictive validity of the UKCAT for medical school undergraduate performance: a national prospective cohort study. BMC Med.

[17] Bala L, Pedder S, Sam AH (2022). Assessing the predictive validity of the UCAT-A systematic review and narrative synthesis. Med Teach.

[18] Hanson JT, Busche K, Elks ML (2022). The Validity of MCAT Scores in Predicting Students’ Performance and Progress in Medical School: Results From a Multisite Study. Acad Med.

[19] Kane MT (1992). An argument-based approach to validity. Psychol Bull.

[20] Cook DA, Brydges R, Ginsburg S (2015). A contemporary approach to validity arguments: a practical guide to Kane’s framework. Med Educ.

[21] Paton LW, McManus IC, Cheung KYF (2022). Can achievement at medical admission tests predict future performance in postgraduate clinical assessments? A UK-based national cohort study. BMJ Open.

[22] Epstein RM, Hundert EM (2002). Defining and assessing professional competence. JAMA.

[23] Norcini JJ, Lipner RS, Kimball HR (2002). Certifying examination performance and patient outcomes following acute myocardial infarction. Med Educ.

[24] Norcini JJ, Boulet JR, Opalek A (2014). The Relationship Between Licensing Examination Performance and the Outcomes of Care by International Medical School Graduates. Academic Medicine.

[25] Tiffin PA, Paton LW, Mwandigha LM (2017). Predicting fitness to practise events in international medical graduates who registered as UK doctors via the Professional and Linguistic Assessments Board (PLAB) system: a national cohort study. BMC Med.

[26] Wakeford R, Ludka K, Woolf K (2018). Fitness to practise sanctions in UK doctors are predicted by poor performance at MRCGP and MRCP(UK) assessments: data linkage study. BMC Med.

[27] Brazeau CMLR, Shanafelt T, Durning SJ (2014). Distress among matriculating medical students relative to the general population. Acad Med.

[28] Department of Health (2017). Expansion of undergraduate medical education: a consultation on how to maximise the benefits from the increases in medical student numbers.

[29] Hsieh FY, Bloch DA, Larsen MD (1998). A simple method of sample size calculation for linear and logistic regression. Stat Med.

[30] McManus IC, Woolf K, Dacre J (2013). The Academic Backbone: longitudinal continuities in educational achievement from secondary school and medical school to MRCP(UK) and the specialist register in UK medical students and doctors. BMC Med.

[31] Ellis R, Brennan P, Scrimgeour DS (2022). Performance at medical school selection correlates with success in Part A of the intercollegiate Membership of the Royal College of Surgeons (MRCS) examination. Postgrad Med J.

[32] Muthén LK, Muthén BO (2002). How to Use a Monte Carlo Study to Decide on Sample Size and Determine Power. Struct Equ Modeling.

[33] Czepiel SA (2002). Maximum likelihood estimation of logistic regression models: theory and implementation, vol. 83.

[34] Montgomery DC, Peck EA, Vining GG (2021). Introduction to linear regression analysis. https://books.google.com/books?hl=en&lr=&id=tCIgEAAAQBAJ&oi=fnd&pg=PR13&dq=Montgomery,+D.C.,+Peck,+E.A.,+%26+Vining,+G.G.+(2021).+Introduction+to+Linear+Regression+Analysis+(6th+ed.).+Wiley.&ots=lgxcZye4Nq&sig=5hzx5wYO6b6wm6bfUd_EbVKnnSU.

[35] Wright S (1934). The Method of Path Coefficients. Ann Math Statist.

[36] Heise DR (1969). Problems in Path Analysis and Causal Inference. Sociol Methodol.

[37] Hu L, Bentler PM (1999). Cutoff criteria for fit indexes in covariance structure analysis: Conventional criteria versus new alternatives. Struct Equ Modeling.

[38] Rubin DB (1976). Inference and missing data. Biometrika.

[39] Little RJA, Rubin DB (2019). Statistical analysis with missing data.

[40] Van Buuren S, Van Buuren S (2012). Flexible imputation of missing data, vol. 10.

[41] McManus IC, Dewberry C, Nicholson S (2013). Construct-level predictive validity of educational attainment and intellectual aptitude tests in medical student selection: meta-regression of six UK longitudinal studies. BMC Med.

[42] White IR, Royston P, Wood AM (2011). Multiple imputation using chained equations: Issues and guidance for practice. Stat Med.

[43] Parliament (1983). Medical act 1983. https://www.legislation.gov.uk/ukpga/1983/54/contents.

[44] HESA Rounding and suppression to anonymise statistics. https://www.hesa.ac.uk/about/regulation/data-protection/rounding-and-suppression-anonymise-statistics.

[45] von Elm E, Altman DG, Egger M (2007). The Strengthening the Reporting of Observational Studies in Epidemiology (STROBE) statement: guidelines for reporting observational studies. Lancet.

