# Supplementary material for: Do University Clinical Aptitude Test (UCAT) scores predict doctors’ performance in UK post-qualification practical clinical examinations? A protocol for an observational retrospective national cohort study
Source: BMJ Open. 2026 Sep 1;16(9):e117049. doi: 10.1136/bmjopen-2026-117049 (PMC13536097; doi:10.1136/bmjopen-2026-117049)
Supplement: Supplementary file 1 [file bmjopen-2026-117049.reviewer_comments.pdf]

PEER REVIEW HISTORY

BMJ Open publishes all reviews undertaken for accepted manuscripts. Reviewers are asked to complete a checklist review form and are provided with free text boxes to elaborate on their assessment. These free text comments are reproduced below.

ARTICLE DETAILS

Title (Provisional)

Do University Clinical Aptitude Test (UCAT) scores predict doctors' performance in UK post-qualification practical clinical examinations? A protocol for an observational retrospective national cohort study.

Authors

Khan, Taha; Paton, Lewis William; Tiffin, Paul Alexander

VERSION 1 - REVIEW

**Reviewer**	**1**
**Name**	**Meier , Niklaus**
**Affiliation**	**Bern University of Applied Sciences, Institute of Health Economics and Health Policy**
**Date**	**05-Feb-2026**
**COI**	**None**

Summary and overall assessment

This protocol describes a study which plans to examine the ability of the University Clinical Aptitude Test (UCAT) to predict post-qualification practical clinical examinations. This research question and relevance of the topic are motivated well in the introduction. In general, the manuscript is very well written and clear. The chosen methods are generally appropriate to answer this research question. I have some comments regarding statistical methodology and terminology. If these comments are addressed, I think this protocol paves the way for a very interesting study.

Major comments

Non-linearity, lines 15 – 53, p. 9: The authors mention the potential for non-linear relationships between predictors and outcomes and hence suggest using random forests to address this. Using random forests is a good idea, but non-linearity could also be integrated into the LASSO regressions by including standardised polynomial terms of continuous variables (x, x^2, x^3, etc.). Since LASSO will automatically remove uninformative variables to determine the parsimonious model, nothing is lost by adding many of these. Similarly, interactions between terms can be added, and irrelevant interaction terms will again be removed by LASSO. A model with many such polynomials and interaction terms can be harder to interpret, but LASSO is in any case suited to maximize predictive power, as its selection of variables has no relation to any causal

interpretation. Please consider this topic and whether polynomials and interaction terms could further improve the LASSO approach in this study.

Prediction vs Causality / Path Analysis, p. 9 – 10 + Figure 1: In general, this protocol describes a study which seeks to estimate how UCAT scores predict clinical examination performance in the data and wisely avoids the topic of causal relationships. Speaking of causality in this context is not illuminating in any case, as the UCAT scores are not a "treatment" and there is no counterfactual scenario to consider. Given the observational nature of the data, there is also no relevant control group in this case, as all students go through the same process. The authors correctly mention that this study is not about causality in the limitations.

Therefore, I am not entirely certain how the authors intend to use their path analysis for the actual statistical analysis. Figure 1 shows a hypothetical causal structure, and as the authors state in the limitations, it is unlikely to be correctly specified based on the underlying data. The causal structure described here shows e.g. an effect of UCAT on the medical school EPM, which has an effect on MSRA CPS, which has an effect on the MRCP PACES. However, this causal structure does not seem valid or useful to me. The UCAT does not have an effect on future test scores but rather captures various factors about the student taking the test. Because it captures these factors, and these factors also impact future test scores, it has potential predictive power. This does not mean that causal effect flows from one test score to the other, and the idea of one test score being a "mediator" for another score seems incorrect to me.

This is a complex topic, and it possible I have misunderstood or misinterpreted something. Overall, my impression is that this study should focus entirely on prediction, and avoid discussions of causality, as the study design and data do not support it. For the same reason, I am sceptical regarding the path analysis. I think this study has value based purely on predictive power, and that causality is not needed. I would like the authors to strongly consider whether the path analysis makes sense as conceived, and either remove it, adapt it, or more convincingly state their case for its validity.

Minor comments

Power analysis, p. 6 (and some other parts of the manuscript): I would recommend avoiding probabilistic statements about hypotheses in frequentist statistics, as interpretations of alpha and beta in this sense are not quite correct. An alpha of 0.05 implies a maximum 5% probability of rejecting a true null hypothesis in repeated sampling. It reflects the probability that the observed data fall in the rejection region, given that the null hypothesis is true, rather than a probability about the null hypothesis. A more careful formulation would be "The alpha (level of statistical significance) was set at 0.05, in line with established practice in medical research, thereby controlling the Type I error rate at 5%." The same holds for the beta, which is defined as as the long-run proportion of failures to reject when the alternative is true. Here a more careful formulation would be "The beta (type II error rate) was set at 0.2, corresponding to a statistical power of 80% under the assumed effect size and significance level." I recommend the authors tighten up the statistical terminology on this topic in the manuscript.

Sample size, line 158, p. 6: The authors write that a sample size of 152 would be sufficient, based on their power analysis. Given the defined the data set and set inclusion/exclusion criteria, I assume that this sample size will easily be reached. If the authors can give some kind of estimate of the likely sample size, this context would be interesting to the reader.

Bias, lines 21 – 27, p. 12: The authors discuss the potential bias from a misspecified causal structure, and then remark "Nevertheless, these assumptions have been mitigated by LASSO and random forests." LASSO and random forests cannot mitigate this risk. As the authors discuss earlier, these methods can potentially help capture non-linear relationships and develop parsimonious models that nonetheless have a high predictive accuracy, without overfitting to the data. But these are separate issues from bias, which LASSO and random forests do not address. Please amend the text to reflect this. Further, if the authors do wish to pursue this direction, though the overall study design is not suited to causal questions (see my comment above), they could look into the "causal forest" method by Athey and Wager.

Abbreviations, figure 1: Figure 1 is missing explanations of the abbreviations used within.

**Reviewer**	**2**
**Name**	**Khanh Le, Nguyen Quoc**
**Affiliation**	**Taipei Medical University**
**Date**	**03-Mar-2026**
**COI**	**None**

---

1. The inclusion criteria require both graduation and attempt at a post-qualification examination. This design conditions on progression through multiple stages (admission, graduation, specialty selection, and exam attempt), each of which may be influenced by the predictors of interest. This creates a serious risk of collider bias, survivorship bias, and additional range restriction. The protocol does not adequately describe how these structural sources of bias will be addressed analytically. Simply acknowledging observational limitations is insufficient. A more explicit discussion of the causal structure and potential bias mechanisms is required.

2. The proposal to use multiple imputation to impute performance in post-qualification examinations for doctors who have not attempted these examinations is highly problematic. Exam performance for non-attempters is not merely "missing" data; it is structurally undefined. The decision to attempt an examination reflects career progression, specialty choice, and timing. Imputing outcomes in this context risks violating missing data assumptions, introducing model-based artefacts, and conflating right-censoring with non-participation. This approach may produce estimates that are difficult to interpret and potentially biased. The authors should reconsider this strategy and provide a more defensible framework for handling non-attempts (e.g., survival analysis, restriction to attempted exams with sensitivity analyses).

3. Incremental validity is primarily operationalised via likelihood ratio tests (logistic models), incremental R2 (linear models). However, statistical significance does not equate to meaningful incremental validity, particularly in large national datasets where small effects are likely to be statistically significant. The protocol does not specify a minimum practically important effect size, decision-relevant thresholds, calibration metrics (e.g., Brier score), and discrimination measures (e.g., AUC).

4. The a priori power calculation is based on a one-sided test under the assumption that negative associations are implausible. While theoretically motivated, this reduces inferential conservatism. In predictive validity research, two-sided testing is standard practice. A clear justification for one-sided testing is required, or the analysis plan should be revised accordingly. Additionally, the power calculation is based on detecting $r = 0.2$, but

no power justification is provided for specialty subgroup analyses, path analysis models, and random forest models.

5. The protocol proposes a wide range of analytic methods: logistic regression, linear regression, LASSO, random forests, path analysis, and multiple imputation. While each method is individually defensible, the protocol lacks a clearly defined primary analysis, a hierarchy of models, a strategy for reconciling conflicting results, and a plan for addressing multiplicity. The inclusion of several exploratory modelling techniques increases the risk of interpretive flexibility and model overfitting. The analytic plan would benefit from simplification and clearer prioritisation.

6. The protocol proposes path analysis to explore mechanisms. However, the data are observational and non-experimental. Key confounders (e.g., socioeconomic factors, non-cognitive traits) may be unmeasured. The causal ordering is assumed rather than demonstrated. Moreover, the proposal to iteratively refine path models based on model fit indices risks post hoc modification and overfitting. The authors should clarify that path models will be exploratory and avoid causal language unless supported by strong assumptions.

7. Range restriction is acknowledged but not formally addressed. Standardisation of scores does not correct for restriction due to selection into medical school or into postgraduate examination attempts. No formal correction (e.g., Thorndike correction) or modelling of the selection process is proposed. This is a central issue in admissions test research and requires stronger treatment.

8. Although some educational predictors are included, the protocol acknowledges that several plausible confounders (e.g., socioeconomic status, clinical exposure, personality traits) cannot be controlled. Given the complexity of educational progression, the authors should consider multilevel modelling (e.g., clustering by medical school), cohort effects, and interaction terms (e.g., specialty by UCAT score).

9. The justification of post-qualification examinations as proxies for clinical competence, while reasonable, is somewhat overstated.

10. Assumption checking for regression models is described in general terms but lacks operational detail.

11. No plan is described for reporting model calibration metrics in logistic regression.

12. The projected timeline for data cleaning and analysis appears optimistic given dataset complexity.

VERSION 1 - AUTHOR RESPONSE

	Feedback	Authors' Response

Summary and overall assessment	Reviewer 1: This protocol describes a study which plans to examine the ability of the University Clinical Aptitude Test (UCAT) to predict post-qualification practical clinical examinations. This research question and relevance of the topic are motivated well in the introduction. In general, the manuscript is very well written and clear. The chosen methods are generally appropriate to answer this research question. I have some comments regarding statistical methodology and terminology. If these comments are addressed, I think this protocol paves the way for a very interesting study.	Thank you for these positive comments.<br><br>We are happy to address the issues raised regarding the statistical methodology and terminology.

|		prospectively on the Open Science Framework (10.17605/OSF.IO/VZ2QC). The planned study dates are from January 2026 to May 2026”.<br><br>The Methods section also now states:<br>“Study Population<br>Since the data source is the UKMED, the population for this study were doctors who graduated from a UK medical school and attempted the updated version of the UCAT, which included SJT, from 2013-2022. The date of extraction was 31st January 2025.”
Outcome justification	Reviewer 2: The justification of post-qualification examinations as proxies for clinical competence, while reasonable, is somewhat overstated.	The Methodical Limitations section now states:<br>“performance during a high-stakes examination may not always reflect behaviour in routine, daily practice.”

Hierarchical analysis	Reviewer 2: The protocol proposes a wide range of analytic methods: logistic regression, linear regression, LASSO, random forests, path analysis, and multiple imputation. While each method is individually defensible, the protocol lacks a clearly defined primary analysis, a hierarchy of models, a strategy for reconciling conflicting results, and a plan for addressing multiplicity. The inclusion of several exploratory modelling techniques increases the risk of interpretive flexibility and model overfitting. The analytic plan would benefit from simplification and clearer prioritisation.	On reflection, we agree that a clearer prioritisation and simplification will strengthen the protocol, providing a more coherent rationale for the analyses proposed. This will also help with the interpretation and potential application of our findings.<br><br>Therefore, the following sentences have been amended to clarify the hierarchal nature of analysis:<br>"Logistic regression will serve as the primary analysis as pass/fail outcomes these are most important for workforce policy since they are instrumental in determining doctors' career progression."<br>"Linear regression will serve as the secondary analysis as continuous relative-to-pass scores provide finer discrimination of performance, complementing the binary pass/fail prediction offered by logistic regression."<br>"Path analysis will serve as the tertiary analysis as it is contingent on findings from the primary logistic and secondary linear regression analyses, which will inform the specification of direct and indirect pathways in the path models. Path analysis also accommodates the temporal ordering and continuity of educational predictors in the model. "<br><br>Our strategy for reconciling potentially conflicting results between our primary and secondary analysis will be narrative, based on the methodological differences in these two approaches.<br><br>While we agree there is value in applying LASSO and random forests on an individual basis, we also accept that, in this context, these additional analyses potentially disrupt the flow and coherence of our analyses. We also acknowledge that the use of these machine learning based techniques increases the risk of overfitting and interpretive flexibility. As such, we have decided to remove these methods from the protocol, and proceed with the methods outlined above.

LASSO	Non-linearity, lines 15 – 53, p. 9: The authors mention the potential for non-linear relationships between predictors and outcomes and hence suggest using random forests to address this. Using random forests is a good idea, but non-linearity could also be integrated into the LASSO regressions by including standardised polynomial terms of continuous variables (x, x^2, x^3, etc.). Since LASSO will automatically remove uninformative variables to determine the parsimonious model, nothing is lost by adding many of these. Similarly, interactions between terms can be added, and irrelevant interaction terms will again be removed by LASSO. A model with many such polynomials and interaction terms can be harder to interpret, but LASSO is in any case suited to maximize predictive power, as its selection of variables has no relation to any causal interpretation. Please consider this topic and whether polynomials and interaction terms could further improve the LASSO approach in this study.	As discussed above, we have removed LASSO from our protocol due to the need to simplify our analytic approach and establish a priori a clear hierarchy of modelling approaches. However, we agree with the reviewer that the LASSO approach would allow for the inclusion of polynomial terms or other non-linear transformations. We thank the reviewer for this point and will consider this in future work.
Path analysis	Reviewer 1: In general, this protocol describes a study which seeks to estimate how UCAT scores predict	Such path models are based on both theory and prior research findings, including our own (e.g. Tiffin et al, 2024a, Tiffin et al, 2024a, Patterson et

	clinical examination performance in the data and wisely avoids the topic of causal relationships. Speaking of causality in this context is not illuminating in any case, as the UCAT scores are not a "treatment" and there is no counterfactual scenario to consider. Given the observational nature of the data, there is also no relevant control group in this case, as all students go through the same process. The authors correctly mention that this study is not about causality in the limitations.<br><br>Therefore, I am not entirely certain how the authors intend to use their path analysis for the actual statistical analysis. Figure 1 shows a hypothetical causal structure, and as the authors state in the limitations, it is unlikely to be correctly specified based on the underlying data. The causal structure described here shows e.g. an effect of UCAT on the medical school EPM, which has an effect on MSRA CPS, which has an effect on the MRCP PACES. However, this causal structure does not seem valid or useful to me. The UCAT does not have an effect on future test scores but rather captures various factors about the student taking the test. Because it captures these factors, and these factors also impact future test scores, it has potential predictive power. This does not mean that causal effect flows from one test score to the other, and the idea of one test score being a "mediator" for another score seems incorrect to me.<br><br>This is a complex topic, and it is possible I have misunderstood or misinterpreted something. Overall, my impression is that this study should focus entirely on prediction, and avoid discussions of causality, as the study design and data do not support it. For the same reason, I am sceptical regarding the path analysis. I think this<br>study has value based purely on	al. 2018). Here, path modelling has been used to delineate the direct and indirect effects of educational predictors and outcomes. Thus, it is appropriate to use this approach in this particular study.<br><br>Path modelling is more suitable to capture and model the temporal ordering of the variables than, say, a simple (2D) multivariable model. For example, educational metrics are recognised to show some degree of continuity ("The Academic Backbone") and therefore their relationship is best modelled in this way.<br><br>We recognise it's a complicated topic and have reworded the text so it can no longer be misconstrued as suggesting causality:<br>"However, a key limitation of path models is that the hypothesised structure needs to be correctly specified in advance of the analysis [Heise, 1969]."

	predictive power, and that causality is not needed. I would like the authors to strongly consider whether the path analysis makes sense as conceived, and either remove it, adapt it, or more convincingly state their case for its validity.<br><br>Reviewer 2: The protocol proposes path analysis to explore mechanisms. However, the data are observational and non-experimental. Key confounders (e.g., socioeconomic factors, non-cognitive traits) may be unmeasured. The causal ordering is assumed rather than demonstrated. Moreover, the proposal to iteratively refine path models based on model fit indices risks post hoc modification and overfitting. The authors should clarify that path models will be exploratory and avoid causal language unless supported by strong assumptions.	
The alpha and beta	Reviewer 1: Power analysis, p. 6 (and some other parts of the manuscript): I would recommend avoiding probabilistic statements about hypotheses in frequentist statistics, as interpretations of alpha and beta in this sense are not quite correct. An alpha of 0.05 implies a maximum 5% probability of rejecting a true null hypothesis in repeated sampling. It reflects the probability that the observed data fall in the rejection region, given that the null hypothesis is true, rather than a probability about the null hypothesis. A more careful formulation would be "*The alpha (level of statistical significance) was set at 0.05, in line with established practice in medical research, thereby controlling the Type I error rate at 5%.*" The same holds for the beta, which is defined as the long-run proportion of failures to reject when the alternative is true. Here a more careful formulation would be "The beta (type II error rate) was set at 0.2, corresponding to a statistical power of 80% under the assumed effect size and significance level." I recommend the authors tighten	We agree and have amended the probabilistic language to:<br>"The alpha (level of statistical significance) was chosen to be 0.05, in line with established practice in medical research, thereby controlling the Type I error rate at a maximum of 5%."<br><br>Similarly, we have also reworded the following:<br>"The beta (Type II error rate) was chosen to be 0.2, corresponding to a statistical power of 80%. This means that, if a true association of the specified magnitude exists, the analyses would be expected to detect it in 80% of repeated samples drawn under the same conditions."<br><br>We have also checked the rest of the manuscript to ensure statistical terminology is used precisely throughout.

	up the statistical terminology on this topic in the manuscript.	
Sample size	Reviewer 1: The authors write that a sample size of 152 would be sufficient, based on their power analysis. Given the defined the data set and set inclusion/exclusion criteria, I assume that this sample size will easily be reached. If the authors can give some kind of estimate of the likely sample size, this context would be interesting to the reader.	In the power analysis section of the Methods, the protocol now states:<br>"This should be comfortably exceeded as the database should provide a sample of at least 250 doctors for the commonly attempted post-qualification practical clinical examinations (see Outcomes)."
One-sided test	Reviewer 2: A clear justification for one-sided testing is required.	In the Power analysis section of the Methods, the protocol states:<br>"A one-sided test, or tail, was used as the theoretical premise [McManus et al., 2013], and existing data [Ellis et al., 2022; Paton et al., 2022], demonstrate that a negative relationship between the predictor and outcome would be very unlikely."<br><br>The theoretical basis for unidirectional prediction rests on three considerations. First, UCAT assesses cognitive abilities, such as verbal reasoning, and situational judgement, that are theoretically aligned with the cognitive and professional competencies assessed in post-qualification clinical examinations. There is no plausible mechanism by which higher cognitive ability or better situational judgement would impair clinical performance. Second, the "academic backbone" framework [McManus et al., 2013] demonstrates that educational attainment exhibits positive correlations throughout the medical training pathway, from secondary school through medical school to post-qualification examinations, with no evidence of ability inversions where higher early performance predicts poorer later performance. Third, the authors are well versed with this field, with significant lived experience too, and have had not come across any published studies examining UCAT and post-qualification clinical examination performance that have reported powered statistically significant negative associations. Therefore, one-sided testing remains the appropriate approach.
Powering additional analyses	Reviewer 2: The power calculation is based on detecting r = 0.2, but no power justification is provided for specialty subgroup analyses, path analysis models, and random forest models.	The study is not powered for subgroup analysis or path analysis. As there was no intention to complete subgroup analysis, it has not been included in the protocol. This has been clarified in the limitations.

		Based on the feedback discussed above, regarding the clarify of analysis and the risk of overfitting, random forests has been dropped from the protocol.<br><br>Power analysis in path models is a complex issue. However, we state that we will consider carrying out a post-hoc power analysis on the final path models, if practical, using Monte Carlo simulation.
Limitations	Reviewer 1: The authors discuss the potential bias from a misspecified causal structure, and then remark "Nevertheless, these assumptions have been mitigated by LASSO and random forests." LASSO and random forests cannot mitigate this risk. As the authors discuss earlier, these methods can potentially help capture non-linear relationships and develop parsimonious models that nonetheless have a high predictive accuracy, without overfitting to the data. But these are separate issues from bias, which LASSO and random forests do not address. Please amend the text to reflect this.	The reference to LASSO and random forests was regarding the other assumptions discussed above. We realise this was confusing. Therefore, as discussed above, based on prior feedback, we have removed LASSO and random forests from the protocol.
Survivorship bias	Reviewer 2: The inclusion criteria require both graduation and attempt at a post-qualification examination. This design conditions on progression through multiple stages (admission, graduation, specialty selection, and exam attempt), each of which may be influenced by the predictors of interest. This creates a serious risk of collider bias, survivorship bias, and additional range restriction. The protocol does not adequately describe how these structural sources of bias will be addressed analytically. Simply acknowledging observational limitations is insufficient. A more explicit discussion of the causal structure and potential bias mechanisms is required.<br><br>Range restriction is acknowledged but not formally addressed. Standardisation of scores does not correct for restriction due to selection into medical -school or into	In this study, survivorship bias is essentially when candidates haven't *survived* the training to attempt the post-qualification practical clinical examinations. This can be for a variety of reasons: they did not gain admission into medical school; they did not pass medical school; they did not complete the pre-requisite post-qualification assessments; they chose not to attempt them (for example they left the UK, left the profession, or postponed progression perhaps due to social circumstances, such as p/maternity leave, or ill-health. This is materially the same as our reference to right censoring, where some doctors had not yet attempted a post-qualification practical clinical examination by the time of analysis.<br><br>Please see below our discussion regarding how we are addressing this censoring and therefore most of the survivorship bias, has been addressed by imputation (see below for further discussion on review feedback regarding our imputations).<br><br>The remaining source of bias in this context, which comes mainly from candidates not gaining admission into medical school, is materially range

	postgraduate examination attempts. No formal correction (e.g., Thorndike correction) or modelling of the selection process is proposed. This is a central issue in admissions test research and requires stronger treatment.	restriction, where candidates with lower UCAT scores have been unsuccessful.<br><br>It is important to correct for this range restriction to establish "construct-level predictive validity" (McManus et al. 2013). It is also important to report the results that have not been corrected for range restriction - as these have implications for policy and practice (see below). Therefore, both sets of results (imputed and non-imputed) should be reported as they have implications for theory and practice, respectively (see also, below).
Imputing outcomes	Reviewer 2: The proposal to use multiple imputation to impute performance in post-qualification examinations for doctors who have not attempted these examinations is highly problematic. Exam performance for non-attempters is not merely "missing" data; it is structurally undefined. The decision to attempt an examination reflects career progression, specialty choice, and timing. Imputing outcomes in this context risks violating missing data assumptions, introducing model-based artefacts, and conflating right-censoring with non-participation. This approach may produce estimates that are difficult to interpret and potentially biased. The authors should reconsider this strategy and provide a more defensible framework for handling non-attempts (e.g., survival analysis, restriction to attempted exams with sensitivity analyses).	This methodological decision was made following extensive consultation with a methodologist specialising in range restriction in personnel selection (author of a doctoral thesis on this topic [https://etheses.whiterose.ac.uk/id/eprint/21267/1/Lazaro_Mwakesi_Mwandigha_PhD_thesis.pdf]). These analyses indicated that, despite its limitations, outcome imputation under MAR assumptions introduced less bias than complete-case restriction or alternative missing data strategies in the context of educational progression data with substantial right censoring.<br><br>Regarding survival analysis as an alternative, variation in examination timing is typically modest (within three years post-qualification) and predominantly reflects factors orthogonal to selection merit (part-time training, parental leave etc.), rather than ability-related delays.<br><br>Instead, we will conduct analyses with and without imputed outcomes. Imputed analysis will inform construct-level predictive validity, whether the constructs (or cognitive abilities) measured by the UCAT subtests predict the doctors' clinical competency (39). In contrast, complete-case analysis of non-imputed data will answer the more policy relevant question, whether the UCAT scores themselves predict the performance of doctors who attempt their post-qualification practical clinical examinations. Comparison between the two estimates reveals the extent to which range restriction attenuates observed correlations. Comparison between the two estimates reveals the extent to which range restriction attenuates observed correlations. This analysis approach avoids the parametric assumptions required for formal range restriction corrections (e.g., Thorndike Case II), which assume both variables

		are normally distributed and that the selection mechanisms are linear, when the latter is especially unlikely to be true, given the multifaceted nature of medical selection.<br>We therefore maintain our analytical approach, whilst providing more robust clarification in the revised manuscript.
Regression	Reviewer 2: Incremental validity is primarily operationalised via likelihood ratio tests (logistic models), incremental R2 (linear models). However, statistical significance does not equate to meaningful incremental validity, particularly in large national datasets where small effects are likely to be statistically significant. The protocol does not specify a minimum practically important effect size, decision-relevant thresholds, calibration metrics (e.g., Brier score), and discrimination measures (e.g., AUC).<br><br>Assumption checking for regression models is described in general terms but lacks operational detail.<br><br>No plan is described for reporting model calibration metrics in logistic regression.	The Power analysis section has been amended to:<br>"The minimum Pearson's r correlation coefficient of practical importance to be accurately detected from the sample was chosen to be 0.2…."<br><br>We have now added a discussion on assumption checking to the Descriptive statistics section:<br>"Q-Q plots will compare the observed data against a theoretical normal distribution, which helps clarify whether the observed data is normally distributed."<br>"Then, a correlation matrix will check for multicollinearity<br>between the variables (see Methodological Limitations)."<br><br>The Regression analysis section has been amended to:<br>"To evaluate the practical importance of the logistic regression findings, the C-statistic ("Area Under the Curve"; AUC) and Number Needed to Reject (NNR) will be reported. The C-statistic is a measure of discrimination that assesses how well the logistic regression model distinguishes between candidates who pass their post-qualification practical clinical examination on their first attempt, from those who fail. It is interpreted as the probability that a randomly selected candidate who passed scored higher on the UCAT than a randomly selected candidate who failed. Thus, a value of 0.5 indicates prediction no better than random chance (50:50), whilst a value of 1.0 indicates perfect discrimination. The NNR is the total number of candidates who would need to be rejected, at a given UCAT score threshold, including those who would have passed their respective post-qualification practical clinical examination, to reject one candidate who would have failed [Tiffin et al., 2016; Tiffin et al., 2017]. Hence, a lower NNR indicates a more effective screening threshold. This is directly relevant to admissions policy, as many universities use UCAT scores as a threshold for inviting candidates to interview. Furthermore, it is more easily

interpretable for our target audience, which is
university selectors and policy makers, who may not have a background in statistics."

VERSION 2 - REVIEW

**Reviewer**	**1**
**Name**	**Meier , Niklaus**
**Affiliation**	**Bern University of Applied Sciences, Institute of Health Economics and Health Policy**
**Date**	**08-May-2026**
**COI**	

The authors have addressed all my comments in a satisfactory manner, and improved the manuscript. I have no further comments, and wish the authors success and all the best in conducting their study.

**Reviewer**	**3**
**Name**	**Dack, Kyle**
**Affiliation**	**University of Bristol, MRC Integrative Epidemiology Unit**
**Date**	**02-Jul-2026**
**COI**	**None**

Statistical review:

This is a clear and thorough protocol. The core analytical methods are strong. However, I have concern regarding the sample size estimation / power analysis. It was calculated for estimating correlations, but this appears to be part of the descriptive analysis and is not the primary analytical method (multivariable regression). Additionally, the section does not have practical impact because this is an analysis of secondary data and there is no control over sample size, so I am unsure whether a power analysis of this kind is really necessary.

A second concern is that it is indicated the multivariable model may be dropped if the univariate model is not significant. The authors established the multivariable model answers a theoretically relevant question, so it is not clear why dropping it would be necessary.

Methods:

1. " The planned study dates are from January to July 2026." As this is a retrospective study, it could be clarified what this refers to - e.g., the dates during which the analysis will be conducted?

2. Power analysis: The justification for not estimating a sample size for path analysis is reasonable, as long as this is taken into account when interpreting the SEM (acknowledging that non-significant paths could reflect inadequate sample size).

-The calculated sample size is based upon a correlation coefficient of 0.2, and is also reasonable.

-My concern is that estimating univariate correlations does not appear to be a primary goal of the study. In the statistical analysis, the correlation analysis is part of the descriptive statistics. This is reflected in the correlation analysis not being mentioned in the abstract at all.

-The primary analysis (based on the abstract, stated aims), appears to be the multivariable regression, as this is what allows estimation of the incremental predictive value (adjusting for educational attainment). This is not discussed in the power analysis, and the section appears somewhat misaligned from the rest of the protocol.

-A more fundamental concern I have is that the power analysis does not have any impact on the study: this is a secondary data analysis, and the sample size is determined only by the availability of data. Power calculations are useful when guiding data collection but in this case it appears somewhat redundant? Especially as the calculation is only for the descriptive element (correlation analysis).

-I suggest the authors consider either performing the sample size calculation using the primary analysis (which appears to be multivariable regression), or removing it because it has no practical impact. Personally I would suggest the latter. [This is also something which could be picked up on in the main paper, if it presents a similar sample size calculation.]

3. Statistical analysis. A thoughtful modelling approach is described which aligns well with the research questions. My only concern is the following: "If the total UCAT z-score demonstrates statistically significant predictive validity in the univariable logistic regression, incremental predictive validity will be assessed using multivariable logistic regression."

This is not ideal, because the primary aim of the study is to assess incremental predictive validity. This is answered by the multivariable model, regardless of whether it is significant or non-significant. By potentially dropping this model, the aim of the study is not fully addressed. Basing the decision on the univariate significance risks introducing data-driven elements to the study and selective reporting, which undermines the theoretical basis of the study.

4. Imputation: This is described thoroughly and clearly. My only concern is the imputation of outcomes. The rationale is not fully clear to me, but this data does not appear "missing" in the strict sense but rather these participants simply did not do the exams, and in that sense

is it not similar to the exclusion criteria of "candidates who attempted the UCAT but did not successfully graduate from medical school". In fact it is unclear how those with missing outcome data differ from this exclusion criteria.

-I suggest the authors consider clarifying this, and possibly as a sensitivity analysis, restrict to those with complete outcome data to quantify what impact this decision has on the results.

VERSION 2 - AUTHOR RESPONSE

Reviewer 1: Dr Niklaus Meier , Bern University of Applied Sciences
The authors have addressed all my comments in a satisfactory manner, and improved the manuscript. I have no further comments, and wish the authors success and all the best in conducting their study.

We thank Dr Meier for their kind words and support in shaping our protocol.

Reviewer 3: Dr Kyle Dack, University of Bristol
This is a clear and thorough protocol. The core analytical methods are strong.

We also thank Dr Dack for their encouraging feedback.

However, I have concern regarding the sample size estimation / power analysis. It was calculated for estimating correlations, but this appears to be part of the descriptive analysis and is not the primary analytical method (multivariable regression).

We agree. Hence, we have and have updated our protocol so that our power analysis aligns with our primary analysis, which is logistic regression. In our amendments in the Power Analysis section, we have clarified our revised approach, which uses the Hsieh, Bloch and Larsen (1998) method.

Additionally, the section does not have practical impact because this is an analysis of secondary data and there is no control over sample size, so I am unsure whether a power analysis of this kind is really necessary.

We agree that power analysis is typically used to inform sample sizes for research. We recognise this can be of limited value when the sample size is fixed, for example in our secondary analysis of an existing dataset. However, the purpose of our power analysis was to support the reader in interpreting our findings. As such, we have reframed this section in terms of the minimum detectable effect size. This will help contextualise the precision of our findings. For example, a non-significant finding for an effect below this benchmark is understood as inconclusive rather than as evidence that no association exists.

A second concern is that it is indicated the multivariable model may be dropped if the univariate model is not significant. The authors established the multivariable model

answers a theoretically relevant question, so it is not clear why dropping it would be necessary.

On reflection, we agree that, counterintuitively, testing for incremental predictive validity should not be contingent on the finding of statistically significant predictive validity. We have amended our plan for regression analyses accordingly, and explained the underlying rationale.

The planned study dates are from January to July 2026." As this is a retrospective study, it could be clarified what this refers to - e.g., the dates during which the analysis will be conducted?

We realise that this wording was potentially misleading and have removed the sentence from the Methods section. The timeline for the study is described in detail in the dedicated Timeline section.

Power analysis: The justification for not estimating a sample size for path analysis is reasonable, as long as this is taken into account when interpreting the SEM (acknowledging that non-significant paths could reflect inadequate sample size).

The limitations of the study, including potentially underpowered path analyses, will most certainly inform the interpretation.

The calculated sample size is based upon a correlation coefficient of 0.2, and is also reasonable. My concern is that estimating univariate correlations does not appear to be a primary goal of the study. In the statistical analysis, the correlation analysis is part of the descriptive statistics. This is reflected in the correlation analysis not being mentioned in the abstract at all.

As discussed above in our response, we agree and have updated the power analysis in our protocol accordingly.

The primary analysis (based on the abstract, stated aims), appears to be the multivariable regression, as this is what allows estimation of the incremental predictive value (adjusting for educational attainment). This is not discussed in the power analysis, and the section appears somewhat misaligned from the rest of the protocol.

As discussed above in our response, we agree and have updated the regression analysis plan in our protocol accordingly.

A more fundamental concern I have is that the power analysis does not have any impact on the study: this is a secondary data analysis, and the sample size is determined only by the availability of data. Power calculations are useful when guiding data collection but in this case it appears somewhat redundant? Especially as the calculation is only for the descriptive element (correlation analysis).

I suggest the authors consider either performing the sample size calculation using the primary analysis (which appears to be multivariable regression), or removing it because it has no practical impact. Personally I would suggest the latter. [This is also something which could be picked up on in the main paper, if it presents a similar sample size calculation.]

As discussed above in our response, we agree and have updated our protocol so that our power analysis aligns with our primary analysis, which is logistic regression.

Statistical analysis. A thoughtful modelling approach is described which aligns well with the research questions. My only concern is the following: "If the total UCAT z-score demonstrates statistically significant predictive validity in the univariable logistic regression, incremental predictive validity will be assessed using multivariable logistic regression."

This is not ideal, because the primary aim of the study is to assess incremental predictive validity. This is answered by the multivariable model, regardless of whether it is significant or non-significant. By potentially dropping this model, the aim of the study is not fully addressed. Basing the decision on the univariate significance risks introducing data-driven elements to the study and selective reporting, which undermines the theoretical basis of the study.

As discussed above in our response, we agree that testing for incremental predictive validity should not be contingent on the finding of statistically significant predictive validity. We have amended our plan for regression analyses accordingly, and explained the underlying rationale.

Imputation: This is described thoroughly and clearly.

We are especially grateful that our description was robust, especially since imputation is a complex and often confusing area.

My only concern is the imputation of outcomes. The rationale is not fully clear to me, but this data does not appear "missing" in the strict sense but rather these participants simply did not do the exams, and in that sense is it not similar to the exclusion criteria of "candidates who attempted the UCAT but did not successfully graduate from medical school". In fact it is unclear how those with missing outcome data differ from this exclusion criteria.

There seem to be two related points here. Firstly, how candidates with incomplete outcome data are different to candidates we excluded who did not successfully graduate medical school. Secondly, the rationale for imputing outcomes.

Regarding the former, if a candidate has not successfully graduated from medical school, they are, by definition, not a qualified doctor. Therefore, they should not be eligible to even attempt the post-qualification practical clinical examinations. Hence, if there is a candidate who has a score for a post-qualification practical clinical

examination, but is not a qualified doctor, those data points are likely to introduce significant bias into our regression models. Conversely, there will be many qualified doctors who have not attempted specific post-qualification practical clinical examinations. This can be for many reasons, such as: they have chosen not to pursue that particular specialty; they have chosen not to attempt those examinations, which are very costly, stressful and time-consuming; or they have not *yet* attempted those examinations, potentially prioritising further post-qualification clinical experience first. Furthermore, there may be doctors who have actually attempted these examinations but have not had their scores saved into this dataset (right censoring).

Regarding the latter, imputing outcome data addresses right censoring, where some doctors had not yet attempted or completed a post-qualification practical clinical examination by the time of analysis, and mitigates range restriction that would occur by excluding these doctors from analysis. It is reasonable to assume that much of the missingness in outcomes (post-qualification clinical examination performance) is missing at random, where it depend on observed variables (such as examination type). However, it could be argued that outcome missingness also has an missingness not at random (MNAR) component: brighter and less risk-averse candidates attempt these examinations earlier in their careers, whilst others delay until they feel adequately prepared due to the high-cost and emotional toll of failing. Nevertheless, even if the MAR assumption does not fully hold, and some degree of MNAR exists, imputation with sensitivity analyses represents the best available approach to address right censoring and range restriction caused by missing outcome data, providing better insights than complete-case analysis alone. Imputed analysis will inform construct-level predictive validity: whether the constructs (or cognitive abilities) measured by the UCAT subtests predict the doctors' clinical competency. In contrast, complete-case analysis of non-imputed data will answer the more policy-relevant question: whether the UCAT scores themselves predict the performance of doctors who attempt their post-qualification practical clinical examinations. Comparison between these two estimates will reveal the extent to which range restriction attenuates observed correlations.

We fully recognise the complexity here. We hope it is sufficiently clear. We would be happy to consider any further changes recommended.

I suggest the authors consider clarifying this, and possibly as a sensitivity analysis, restrict to those with complete outcome data to quantify what impact this decision has on the results.

Since we are already comparing the estimates between complete case and fully imputed (predictors and outcomes) analysis, we can include a further sensitivity analysis where we compare the estimates after imputing the predictors but not the outcomes. We have added this to the protocol and look forward to the feedback
